# Early de-escalation of antifungal treatment in critically ill immunocompromised patients with proven or probable invasive candidiasis: A retrospective multicentre study

**DOI:** 10.1016/j.aicoj.2026.100139

**Published:** 2026-08-25

**Authors:** Karim Jaffal, Constance Bayon, Louis Kreitmann, Anahita Rouzé, Marie Labruyere, Annabelle Stoclin, Jean Reignier, Guillaume Brunin, Anais Dartevel, Boualem Sendid, Julien Labreuche, Saad Nseir

**Affiliations:** a Infectious Disease Department, R Poincaré University Hospital, Garches, APHP, Versailles Paris Saclay University, France; b CHU Lille, Service de Médecine Intensive Réanimation, F-59000 Lille, France; c Critical Care, Guy's and St Thomas' NHS Foundation Trust, London, United Kingdom; d Centre for Critical Illness Research, School of Immunology and Microbial Sciences (SIMS), King's College London, Guy's Hospital, London, United Kingdom; e Univ. Lille, U1285 - UMR 8576 - UGSF - Unité de Glycobiologie Structurale et Fonctionnelle, F-59000 Lille, France; f CNRS, UMR 8576, F-59000 Lille, France; g Inserm, U1285, F-59000 Lille, France; h Médecine Intensive - Réanimation, Centre Hospitalier Universitaire de Dijon, 14 Rue Gaffarel, BP 77908, 21079 Dijon Cedex, France; i Gustave Roussy, Service de réanimation et de Soins Intensifs, 94805 Villejuif, France; j Medecine Intensive Réanimation, Centre Hospitalier Universitaire de Nantes, France; k Intensive Care Unit, CH de Boulogne-sur-Mer, France; l Medical Intensive Care Unit, Grenoble Alpes University Hospital, Grenoble, France; m Laboratory of Mycology and Parasitology, CHU Lille, 59000 Lille, France; n Department of Biostatistics, CHU Lille, Lille, France

**Keywords:** Immunocompromised patients, Critically ill patients, Invasive fungal infection, Invasive candidiasis, Antifungal treatment

## Abstract

**Background and objectives:**

Antifungal treatment (AFT) for proven or probable invasive candidiasis (IC) is common in critically ill immunocompromised patients. Early AFT de-escalation appears safe in unselected ICU patients, but has not been studied specifically amongst immunocompromised hosts.

**Methods:**

This retrospective study was conducted in 19 ICUs in France between January 2019 and December 2024. All immunocompromised patients hospitalized in ICU for ≥5 days and treated with antifungals for a first proven or probable episode of IC were included. Early AFT de-escalation was defined as either spectrum reduction, i.e., a switch from an initial echinocandin or liposomal amphotericin B to fluconazole, or complete discontinuation of the initial antifungal, occurring within 5 days (day 1–5) of treatment initiation. The primary objective was to determine the incidence of early AFT de-escalation (censored at day 28 after initiation of AFT).

**Results:**

242 patients were analysed (median age 64 years, 64% males). Most patients (*n=*175, 72.3%) were treated empirically or pre-emptively for probable IC. Early AFT de-escalation occurred in 92 patients (38%, 95% confidence interval [CI] 31.9%–44.2%). AFT de-escalation consisted of spectrum reduction in 35.9%, and AFT discontinuation in 64.1% of cases. There was no difference in ICU mortality, ICU length-of-stay and the number of ventilator-free days between patients with and without AFT de-escalation. AFT was re-initiated within 7 days of de-escalation in 6 of the 92 patients (6.5%) who had undergone early AFT de-escalation.

**Conclusions:**

Early AFT de-escalation occurred in 38% of critically ill immunocompromised patients, and was not associated with adverse prognostic outcomes.

## Introduction

In the last decades, the survival of patients with cancer, hematologic malignancies and autoimmune diseases has increased thanks to the availability of new treatments. Because of parallel improvements in the care of critically ill patients, their outcomes following an ICU admission have also improved, and immunocompromised patients now account for a substantial part of the typical ICU case-mix [1,2]. However, these patients remain particularly susceptible to ICU-acquired infections [3].

Amongst these, invasive candidiasis (IC)—which can manifest as candidemia or deep-seated (visceral) candidiasis—is the most frequent fungal infection [4] and poses important challenges in management [5]. First, the clinical manifestations of IC are highly non-specific. Second, diagnostic tools have limited diagnostic performance (low sensitivity and/or specificity) and suffer from a long turnaround time (especially culture) [6]. Third, the mortality of IC is particularly high: 40% in unselected cases [7], and up to 60% when associated with septic shock [8]. Finally, delay between IC onset and initiation of adequate antifungal treatment (AFT) has been associated with an increased mortality [9]. To mitigate the risk of delayed or missed treatment, early empirical AFT against *Candida* is recommended for patients with septic shock or signs of deterioration who have risk factors for IC (including immunosuppression), before the results of culture establish the diagnosis of proven IC [10,11]. While the impact of this strategy on mortality has not been formally established [12], even for patients with septic shock [13], early empirical AFT accounted for around two thirds of all AFT prescriptions in the ICU a decade ago [14]. The main challenge after AFT initiation in this context is to decide when to stop AFT, for instance because another cause or source of infection is identified, or because biomarkers of IC with high negative predictive value are negative [15,16].

Irrespective of whether AFT is initiated for proven or probable IC, echinocandins are the recommended first-line agents [10,11,17]. In the context of proven IC in non-neutropenic patients, the European Society for Clinical Microbiology and Infectious Diseases (ESCMID) and the Infectious Diseases Society of America (IDSA) guidelines recommend a de-escalation strategy whereby echinocandins are replaced with fluconazole when the patient is clinically stable, has fluconazole-susceptible isolates, and negative blood cultures. The recommended timing of AFT de-escalation is after 5 days of clinical stability for IDSA [10], and 10 days overall for ESCMID [17]. However, these recommendations are based on low-level evidence, and do not apply to immunocompromised patients. The more recent 2025 guidelines by the European Confederation for Medical Mycology (ECMM), the International Society of Human and Animal Mycology (ISHAM) and the American Society for Microbiology (ASM) suggest a switch to an oral azole after at least 5 days of echinocandin treatment in stable, non-neutropenic patients with clearance of *Candida* from the bloodstream, appropriate source control and confirmed susceptibility to the chosen azole [11].

AFT de-escalation encompasses different clinical situations depending on whether AFT has been initiated for proven or probable IC: spectrum reduction, i.e., the switch from an initial antifungal (usually an echinocandin) to fluconazole in proven IC [18,19], and complete discontinuation of empirical AFT in probable IC when the diagnosis is ultimately excluded [15,16,20,21]. In all cases, reducing AFT use in the ICU appears as an important component of antifungal stewardship, as unjustified AFT can promote the emergence and spread of antifungal-resistant *Candida* strains [22] and is associated with higher costs and potential drug toxicity [23]. The safety of AFT de-escalation in proven and probable IC has been suggested by observational and interventional studies [15,16,[18], [19], [20], [21]], but has not been investigated specifically amongst immunocompromised patients. Therefore, the aim of this study was to determine the incidence of AFT de-escalation in a multicentre cohort of immunocompromised patients, and assess its association with outcomes.

## Methods

### Study design, setting, patients

This retrospective observational study was conducted from January 2019 to December 2024 in 19 ICUs of tertiary-care teaching hospitals in France.

All immunocompromised patients hospitalized for more than 5 days in the ICU who required systemic AFT for a first episode of proven or probable IC were eligible. Immunosuppression was defined as: solid cancer (active or in remission for less than 5 years), hematologic malignancy (active or in remission for less than 5 years), solid-organ transplant, long-term (≥28 days) use of steroids (≥10 mg of prednisone per day or equivalent) or other immunosuppressant drugs, human immunodeficiency virus (HIV) infection with <200 CD4 + T cells/mm^3^ (or unknown) [24,25].

Patients receiving prophylactic AFT, patients with an ICU length-of-stay ≤5 days (those who either died or were discharged before day 5) and patients with a proven or probable mold infection were excluded.

### Definitions of invasive candidiasis, antifungal treatment indications and de-escalation

Proven and probable IC cases were defined according to the criteria of the European Organization for Research and Treatment of Cancer/Invasive Fungal Infections Cooperative Group (EORTC) published in 2008 [26] and updated in 2021 [27]. The diagnosis of *proven IC* required the demonstration of fungal elements by histopathology or the recovery of *Candida* by culture from a normally sterile site, irrespective of host factors or clinical features. The diagnosis of *probable IC* was based on the presence of host factors (immunosuppression, impaired gut wall or skin barrier integrity, multifocal colonization), compatible clinical findings (compatible ocular findings by fundoscopic examination, hepatosplenic lesions by computed tomography [CT], clinical or radiological abnormalities consistent with an infectious-disease process that are otherwise unexplained) and mycological evidence (including the positivity of serum (1,3)-β-d-glucan in two consecutive samples or positive *Candida* cultures not fulfilling criteria for proven IC).

Indications for AFT were defined according to the ESICM/ESCMID task force on the practical management of IC in critically ill patients [28]. *Documented treatment* (also called directed or targeted therapy) was defined as AFT initiated in the context of proven IC. *Empirical treatment* referred to the initiation of AFT in patients at high risk for IC who had clinical signs of infection (such as persistent fever or septic shock), but no microbiological criteria fulfilling the definition of proven IC. *Pre-emptive treatment* referred to patients who had risk factors for IC, no clinical signs of infection, and positive biomarkers (usually (1,3)-β-d-glucan, mannan antigen or anti-mannan antibodies).

*Early AFT de-escalation* was defined as either *spectrum reduction*, i.e., a switch from an initial echinocandin or liposomal amphotericin B to fluconazole, or *complete discontinuation* of the initial antifungal, occurring within 5 days (day 1–5) of treatment initiation. The cut-off of 5 days was chosen arbitrarily to align our study with prior observational studies and guidelines on the topic [10,11,20,21]. All initiation and de-escalation of AFT, and choices of molecules and doses were left at the discretion of attending physicians, following local guidelines derived from international guidelines [10,17,28].

*AFT re-initiation* was defined as the re-initiation of any systemic AFT within 7 days of its discontinuation (for any reason).

### Study objectives

The primary objective was to determine the proportion of immunocompromised critically ill patients alive and remaining in ICU 5 days after AFT initiation who underwent early AFT de-escalation. Secondary objectives were to assess the association of AFT de-escalation with ICU mortality, ICU length-of-stay, the number of days alive without invasive mechanical ventilation (IMV-free days — all outcomes censored at 28 days after AFT initiation). We also reported the proportion of *AFT re-initiation* amongst patients who had undergone AFT de-escalation.

### Data collection

Clinical data were retrospectively recorded from archived healthcare records at each participating center. Only the first episodes of proven or suspected IC were considered. Microbiological data were collected for each patient from the mycology laboratory, and pharmacy records were used to extract details on antifungal prescription at each participating center. The Candida score was calculated for each patient according to reference [29]. Multifocal colonization required isolation of *Candida* spp. from at least two non-srerile sites (including respiratory tract [endotracheal aspirate or bronchoalveolar lavage], urine, skin/wounds, and gastrointestinal tract [i.e., stool or rectal swab]) within a 7-day period. Serum (1–3)-β-d-glucan was measured using the Fungitell® assay (Associates of Cape Cod Inc., Falmouth, MA, USA), with a cut-off value of 80 pg/mL to define positivity. Patients were followed up until day 28 after AFT initiation or until ICU discharge (whichever came first).

### Statistical analysis

Quantitative variables were presented as means (standard deviation) or medians (interquartile range [IQR], i.e., 25th to 75th percentiles) according to the normality of distribution, while categorical variables were expressed as numbers (percentage). The normality of distributions was assessed using histograms and the Shapiro–Wilk test.

Patient characteristics at ICU admission and characteristics of IC episodes and antifungal management during ICU stay were described and compared between patients with and without early AFT de-escalation. Quantitative variables were compared using Student’s *t*-test or Mann-Whitney *U* test depending on the normality of distribution, and categorical variables were compared using the Chi-square test, or Fisher’s exact test in cases of expected cell frequencies <5.

Factors associated with AFT de-escalation in univariable analyses (*p* < 0.20) were included in a multivariable logistic regression model. Odds ratios (ORs) and their 95% confidence intervals (CIs) were reported as measures of association. ICU mortality and ICU length-of-stay were described according to AFT de-escalation status using a survival analysis approach, by estimating the cumulative incidence of the event of interest (ICU death and ICU discharge alive, respectively) and considering respectively ICU discharge alive and ICU death as competing events, as appropriate. Cumulative incidences were estimated using the Aalen–Johansen estimator, starting on the 5th day after AFT initiation, until the 28th day after AFT initiation or ICU discharge, whichever occurred first, and were compared using Gray’s test.

The impact of early AFT de-escalation on these two outcomes was assessed using cause-specific Cox proportional hazards models (because our aim was to assess etiological associations [30,31]). Cause-specific hazard ratios (cHR) were reported as effect sizes, a cHR > 1.0 for ICU mortality being associated with a higher risk of ICU death, and a cHR > 1.0 for ICU discharge alive being associated with a shorter ICU length-of-stay. Proportional hazard assumptions were assessed using scaled Schoenfeld residuals plots. Between-group comparisons were further adjusted on clinically relevant confounders, namely age, sex, Simplified Acute Physiology Score (SAPS)-II score, post-operative care and sepsis or shock during ICU stay. Finally, we compared the number of 28-day ventilator-free days between patients with and without AFT de-escalation using Mann-Whitney *U* test. Effect sizes were expressed as generalized odds ratios (genOR) by splitting ties equally between both groups. Adjusted GenORs were obtained using the inverse probability of treatment weighting (IPTW)-weighted win odds ratio method [32], a genOR>1.0 being associated with higher ventilator-free days. We further assessed potential heterogeneity in the association of AFT de-escalation with outcomes according to proven and probable IC by estimating unadjusted effect sizes within each subgroup. Finally, we assessed the effect of AFT de-escalation on outcomes by differentiating the type of AFT de-escalation (spectrum reduction vs. complete discontinuation); unadjusted effect sizes for ‘spectrum reduction’ and ‘complete discontinuation’ versus ‘no de-escalation’ were estimated as main analysis.

Statistical testing was done at the two-tailed α level of 0.05. Data were analysed using the SAS software package, release 9.4 (SAS Institute, Cary, NC, USA) and using the WINS package (version 1.4.3) of the R software, version 4.1.2 (R Foundation for Statistical Computing, Vienna, Austria).

### Ethics

The Ethics Committee and Institutional Review Board of the Lille University Hospital approved the study protocol (*Comité de Protection des Personnes Ouest III*; approved in August 2019, registration number 2017-A03113-50). Because of the retrospective observational design of the study, and in accordance with French law, written informed consent was not required by the local institution. However, patients or their relatives were informed about their participation in the study and were given the possibility to refuse.

## Results

### Patient characteristics at ICU admission

Between January 2019 and December 2024, 242 patients from 19 ICUs were analysed (median age 64, IQR 53–70 years, 64% males — Table 1). The main causes of immunosuppression were treatment with steroids and/or immunosuppressants (*n* = 116), hematologic malignancy (*n* = 104) and solid cancer (*n* = 83). Sepsis was the leading cause of ICU admission.

**Table 1:** Patients’ characteristics at ICU admission.

Characteristics	AFT de-escalation		
	No (*n* = 150)	Yes (*n* = 92)	*P-V*alue
Age, years, median (25th to 75th percentiles)	63 (53–69)	66 (54–73)	0.047
Men	90/150 (60.0)	65/92 (70.7)	0.094
SOFA	7.9 (3.8)	7.8 (3.9)	0.83
SAPS II	51.4 (19.2)	52.7 (19.4)	0.62
**Comorbidities**			
Diabetes	28/150 (18.7)	21/92 (22.8)	0.43
Chronic respiratory failure	5/150 (3.3)	5/92 (5.4)	0.51
COPD	15/150 (10.0)	11/92 (12.0)	0.63
Chronic heart failure	9/150 (6.0)	4/92 (4.3)	0.77
Liver cirrhosis	10/150 (6.7)	2/92 (2.2)	0.14
Chronic renal disease	10/150 (6.7)	9/92 (9.8)	0.38
Recent hospitalization (within 3 months of ICU admission)	101/149 (67.8)	69/92 (75.0)	0.23
Prior antibiotic treatment (within 1 month of ICU admission)	88/149 (59.1)	48/92 (52.2)	0.30
Prior antifungal treatment (within 1 month of ICU admission)	15/148 (10.1)	7/92 (7.6)	0.51
**Immunosuppression**			
Solid malignancy	46/150 (30.7)	37/92 (40.2)	0.13
Hematologic malignancy	67/150 (44.7)	37/92 (40.2)	0.50
Steroids and/or immunosuppressive therapy	68/150 (45.3)	48/92 (52.2)	0.30
Solid organ transplant	11/150 (7.3)	6/92 (6.5)	0.81
HIV (with CD4 + T cells< 200/mm^3^ or unknown)	2/150 (1.3)	0/92 (0.0)	NA
**Transfer from**			
Home	23/150 (15.3)	16/92 (17.4)	0.89
Other wards	118/150 (78.7)	70/92 (76.1)	
Other ICUs	9/150 (6.0)	6/92 (6.5)	
**Admission category**			
Medical	119/150 (79.3)	65/92 (70.7)	0.16
Scheduled surgery	11/150 (7.3)	6/92 (6.5)	
Urgent surgery	20/150 (13.3)	21/92 (22.8)	
**Causes for ICU admission**			
Acute respiratory failure (any)	68/150 (45.3)	43/92 (46.7)	0.83
Acute respiratory distress syndrome	39/150 (26.0)	29/92 (31.5)	.
Acute COPD exacerbation	2/150 (1.3)	0/92 (0.0)	
Community-acquired/nosocomial pneumonia	21/150 (14.0)	10/92 (10.9)	.
Congestive heart failure	1/150 (0.7)	2/92 (2.2)	.
Other(s)	6/150 (4.0)	2/92 (2.2)	.
Shock (any)	80/150 (53.3)	61/92 (66.3)	0.047
Septic shock	61/150 (40.7)	55/92 (59.8)	.
Cardiogenic shock	1/150 (0.7)	0/92 (0.0)	.
Hypovolemic shock	16/150 (10.7)	5/92 (5.4)	.
Other	2/150 (1.3)	1/92 (1.1)	.
Sepsis	121/150 (80.7)	75/92 (81.5)	0.87
Neurologic failure	23/150 (15.3)	15/92 (16.3)	0.84
Acute kidney injury	50/150 (33.3)	33/92 (35.9)	0.69
Acute liver failure	10/150 (6.7)	5/92 (5.4)	0.70
Post-operative care	33/150 (22.0)	32/92 (34.8)	0.029
Other(s) cause of ICU admission	6/150 (4.0)	5/92 (5.4)	0.75

Values are no./total no. (%) or mean (standard deviation) unless otherwise as indicated.

Abbreviations: COPD = chronic obstructive pulmonary disease; ICU = intensive care unit; SAPS-II: Simplified Acute Physiology Score II; SOFA: sequential organ failure assessment.

### Characteristics of IC episodes and initial antifungal management

AFT was predominantly initiated empirically (63%), with a smaller proportion of treatments started in documented (28%) or pre-emptive (9%) settings (Table 2). Echinocandins, mainly caspofungin (87%), were used as first-line AFT. Patients were severely ill, with a high prevalence of IMV (77%), and sepsis or septic shock (92%). The median Candida score was 3, and classical risk factors for IC were common. Approximately half of the patients underwent (1,3)-β-d-glucan testing (48%), with a substantial proportion yielding positive results (53%). Most episodes were classified as probable IC (*n* = 171, 71%), while 71 episodes (29%) were proven. Amongst patients with proven IC, candidemia was the most frequent presentation (39/71, 54.9%), followed by deep-seated candidiasis (28/71, 39.4%), with no significant difference in IC localisation according to AFT de-escalation status (*p* = 0.322). *Candida albicans* was the leading causative species amongst proven IC cases and was more frequently isolated from sterile sites in patients undergoing AFT de-escalation than in those without de-escalation (82.6% vs. 54.2%; *p* = 0.028), whereas *Nakaseomyces glabratus* was less frequent (4.3% vs. 27.1% — Supplementary Table 1). Concomitant bacterial infections were frequently documented (73%).

**Table 2:** Characteristics of invasive candidiasis episodes and antifungal management during ICU stay.

Characteristics	AFT de-escalation		*P-Va*lue
	No (*n* = 150)	Yes (*n* = 92)	
**AFT indication**			0.56
Documented	44/150 (29.3)	23/92 (25.0)	
Empirical	91/150 (60.7)	62/92 (67.4)	
Pre-emptive	15/150 (10.0)	7/92 (7.6)	
**First-line AFT**			0.71
Caspofungin	128/150 (85.3)	83/92 (90.2)	
Amphotericin B	9/150 (6.0)	5/92 (5.4)	
Fluconazole	10/150 (6.7)	3/92 (3.3)	
**Organ failures**			
Invasive mechanical ventilation	115/149 (77.2)	70/90 (77.8)	0.92
Renal replacement therapy	46/150 (30.7)	23/92 (25.0)	0.34
**IC risk factors and management**			
Time between ICU admission and AFT initiation	3 (1–12)	3 (1–9)	0.54
Total duration of AFT treatment			
Amongst patients alive at AFT discontinuation^a^	10 (7–15) [*n* = 126]	5 (3–9) [*n* = 90]	ND
Amongst patients who died before AFT discontinuation	14 (11–20) [*n* = 24]	(20; 27) [*n* = 2]	ND
Antibiotic treatment	144/150 (96.0)	89/92 (96.7)	1.00
Candida score	3.0 (2.0–4.0)	3.0 (2.0–4.0)	0.069
Parenteral nutrition	76/150 (50.7)	45/92 (48.9)	0.79
Surgery at admission	41/150 (27.3)	33/92 (35.9)	0.16
Multifocal *Candida* colonization^b^	37/150 (24.7)	29/92 (31.5)	0.25
Sepsis or septic shock	133/150 (88.7)	89/92 (96.7)	0.027
Removal of central venous catheter	79/150 (52.7)	41/92 (44.6)	0.22
Severe neutropenia (neutrophils<0.5 G/L)	33/109 (30.3)	13/71 (18.3)	0.072
**(1,3)-β-d-glucan**			
Dosage performed	79/150 (52.7)	37/92 (40.2)	0.060
Positive (1,3)-β-d-glucan^c^	45/79 (57.0)	16/37 (43.2)	0.17
**Type of IC**			0.25
Proven	48/150 (32.0)	23/92 (25.0)	
Probable	102/150 (68.0)	69/92 (75.0)	
**Localisation of IC (amongst patients with proven IC)**			0.32
Candidemia	24/48 (50.0)	15/23 (65.2)	
Deep-seated candidiasis	20/48 (41.7)	8/23 (34.8)	
Deep-seated candidiasis + candidemia	4/48 (8.3)	0/23 (0)	

Values are no./total.no (%) or median (25th to 75th percentiles) [number of available cases]. ND indicates not done.

AFT: antifungal therapy; IC: invasive candidiasis; ICU: intensive care unit.

a Including 6 patients under AFT at day 28 after AFT initiation (5 in no AFT de-escalation and 1 in AFT de-escalation).

b Defined as ≥2 positive *Candida* spp. cultures at non-blood sites.

c A positive (1,3)-β-d-glucan was defined as a value ≥80 pg/mL, according to manufacturer instructions.

### Incidence and factors associated with early AFT de-escalation

Overall, early AFT de-escalation was performed in 92 of 242 episodes, corresponding to an incidence of 38% in the whole cohort (95% CI 31.9–44.2%). Amongst these de-escalation episodes, 69 (75%) occurred in patients classified as having probable IC, whereas 23 episodes (25%) occurred in proven IC (Table 2). Amongst the 171 patients with probable IC, 69 (40.4%, 95% CI 33.0–47.8%) underwent early AFT de-escalation. Similarly, early AFT de-escalation was more frequently observed in patients initially treated empirically (67.4%) than in those receiving documented treatment (25.0%). However, neither the initial indication for AFT, nor the proportion of proven and probable IC differed significantly between the de-escalation and no de-escalation groups.

Amongst the 92 patients who underwent early AFT de-escalation, the initial AFT was switched to an azole in 33 patients (spectrum reduction, 36%) and discontinued in 59 patients (64%). De-escalation occurred at a median of 4 days (IQR 3–5) after AFT initiation. Amongst patients alive at AFT discontinuation, the median treatment duration was shorter in the de-escalation group than in the non-de-escalation group (5 days [IQR 3–9] vs. 10 days [IQR 7–16]). The main reasons for de-escalation reported by treating physicians were a documented bacterial infection (39%), susceptibility to a narrower-spectrum antifungal based on susceptibility testing (27%), negative *Candida* cultures (14.1%), negative (1,3)-β-d-glucan results (8.7%), and recovery from neutropenia (2.2%). Of note, a local protocol guiding initiation or de-escalation of AFT was reported in fewer than 5% of cases.

Patients with early AFT de-escalation were significantly older and had a higher proportion of shock at ICU admission than patients without AFT de-escalation (Table 1). Other demographic characteristics, comorbidities, prior healthcare exposure, causes of immunosuppression, admission severity scores, or reasons for ICU admission were not significantly different between groups. No significant independent predictor of early AFT de-escalation was identified in multivariate analysis. Only male sex (OR = 1.73, 95% CI, 0.97–3.10) and shock (OR = 1.67; 95% CI 0.93–3.00) were associated with higher odds of de-escalation, although both CIs included 1 (Supplementary Table 2). Patients in the de-escalation group had a higher rate of sepsis or septic shock (97% vs. 89%, *p* = 0.027) and a higher frequency of concomitant proven bacterial infection (82% vs. 67%, *p* = 0.016) during ICU stay. No significant differences were observed regarding Candida score, classical risk factors for IC, (1,3)-β-d-glucan testing and positivity, or central venous catheter removal.

### Patient outcomes

**Fig. 1:**
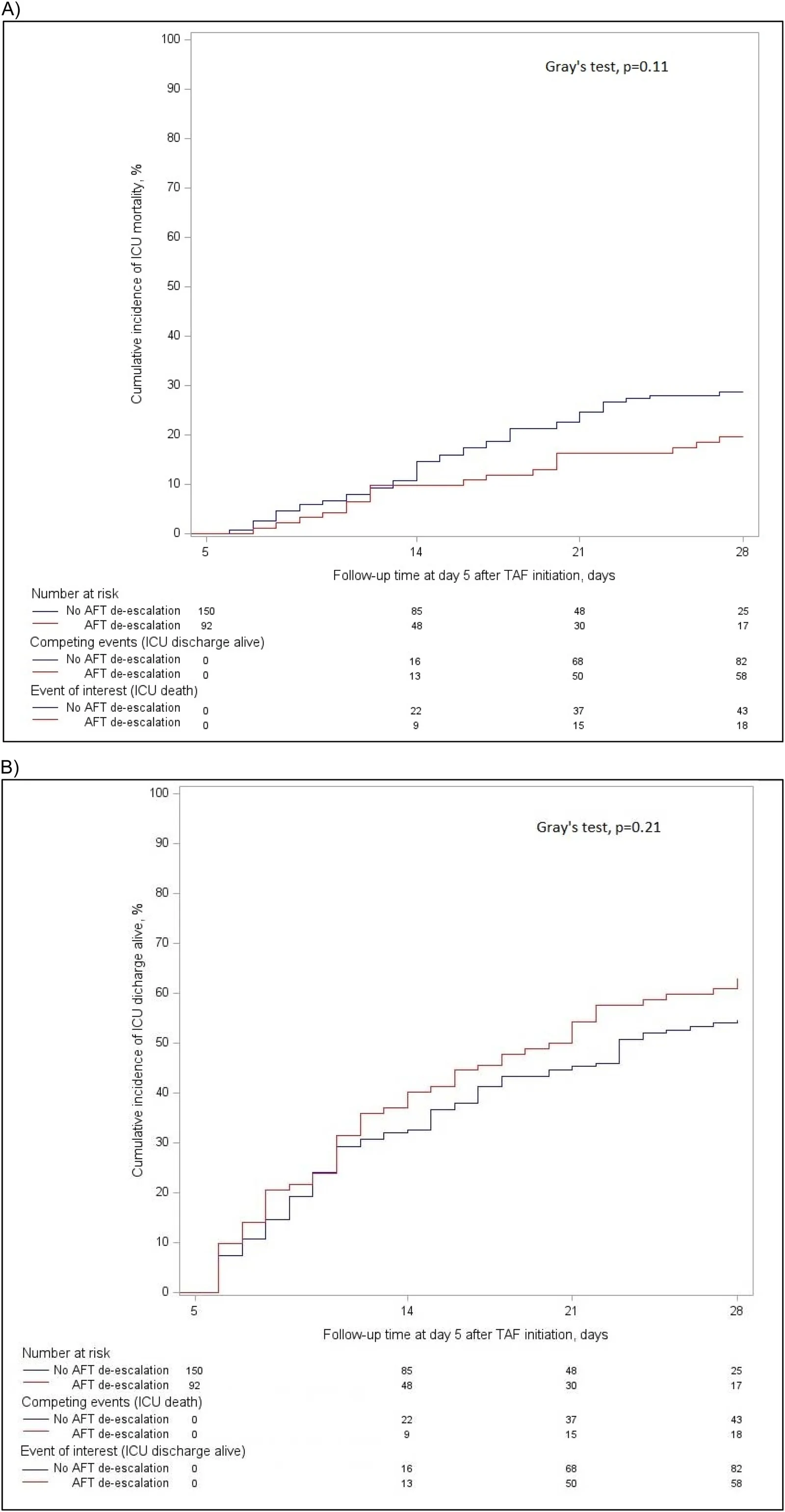
Cumulative incidence of ICU mortality (considering ICU discharge alive as a competing event) (A) and ICU discharge alive (considering ICU death as a competing event) (B), according to AFT de-escalation status at day 5.

The cumulative incidences of ICU mortality and of ICU discharge alive are reported in Fig. 1. In univariate and multivariate cause-specific Cox regression models (treating ICU discharge alive and ICU mortality as competing events, respectively), early AFT de-escalation was not significantly associated with an increased risk of ICU mortality or with a longer ICU length-of-stay (Table 3). We also found no between-group differences in IMV-free days. There was no significant difference in outcomes according to the type of AFT de-escalation (spectrum reduction vs. complete discontinuation — Supplementary Table 3), or amongst patients with proven vs. probable IC (Supplementary Fig. 1). Early AFT re-initiation was observed in 6 of 92 patients (6.5%) who had undergone AFT de-escalation.

**Table 3:** Patients’ outcomes.

28-day outcomes	AFT de-escalation		Unadjusted		Adjusted^a^	
	No (*n* = 150)	Yes (*n* = 92)	Effect size (95%CI)	p-value	Effect size (95%CI)	p-value
ICU mortality			0.67 (0.38–1.17)	0.16	0.62 (0.35–1.10)	0.10
no./total no. (%)	43/150 (28.7%)	18/92 (19.6%)				
incidence rate (%) per exposure days	43/2439 (1.7)	18/1506 (1.1)				
Ventilator-free days	16 (0–26) [*n* = 149]	17 (2–25) [*n* = 90]	1.09 (0.81–1.48)	0.55	1.08 (0.80–1.45)	0.62
ICU length-of-stay			1.14 (0.81–1.61)	0.43	1.17 (0.83–1.66)	0.37
Incidence rate (%) per exposure days	82/2439 (3.4)	58/1506 (3.6)				
Alive patients, median (25th to 75th percentiles)	15 (9–27)	15 (8–27)				
Dead patients, median (25th to 75th percentiles)	14 (11–20)	14 (11–20)				

Incidence of ICU death or ICU discharge alive calculated per exposure day.

Effect sizes were assessed using two different measures: 1) the cause-specific hazard ratio (cHR), treating ICU discharge alive (for the ‘ICU mortality’ outcome) and ICU mortality (for the ‘ICU length-of-stay’ outcome) as competing risk; 2) generalized odds ratio provided for higher ventilator-free days values.

ICU: intensive care unit.

a Adjusted for age, sex, SAPS-II, post-operative care and sepsis or shock during ICU stay.

## Discussion

In this retrospective multicentre study, early AFT de-escalation was performed in 38% of critically ill immunocompromised patients receiving documented, empirical or pre-emptive AFT for proven or probable IC.

In previous studies conducted amongst unselected ICU patients, the incidence of AFT de-escalation was generally lower: 20% in a retrospective monocentric study by our group (*n* = 190) [20], and 22% in an ancillary analysis of a multicentre prospective cohort (*n* = 647) [21]. This could reflect differences in the proportion of proven vs. probable IC across studies. In this cohort, the proportion of patients with proven IC was lower, and consequently the proportion of patients in whom AFT was started empirically or pre-emptively was higher, than in previous studies. It can be hypothesized that intensivists have a lower threshold to start empirical/pre-emptive AFT in immunocompromised than in non-immunocompromised patients, and that in this context they are subsequently more likely to interrupt AFT when IC is ruled out (for instance because bacterial cultures become positive or fungal biomarkers return negative).

While we previously found associations between indications for AFT (i.e., pre-emptive vs. documented or empirical), multifocal *Candida* colonization, certain treatments received during ICU stay (including IMV) and AFT de-escalation [20], in this cohort the main factor associated with AFT de-escalation was the positivity of bacterial cultures. However, reasons for de-escalation reported by attending physicians also included the negativity of *Candida* cultures and fungal biomarkers. There was also a trend towards a higher proportion of neutropenic patients amongst those without AFT de-escalation, suggesting that clinicians may be more cautious in de-escalating therapy in patients with profound immunosuppression. The higher proportion of *C. albicans* and lower proportion of *N. glabratus* amongst patients undergoing AFT de-escalation may also reflect clinicians’ greater willingness to narrow treatment when a species with expected fluconazole susceptibility is identified, although antifungal susceptibility patterns were not systematically analysed in our study.

The focus of this study on immunocompromised patients with proven or probable IC was justified by the fact that these patients account for a growing proportion of ICU admissions, and that decisions to de-escalate AFT may be particularly challenging given the risks associated with inadequate AFT in this vulnerable population. However, we acknowledge the substantial heterogeneity within this cohort, in terms of types, severity and duration of immune defects, IC susceptibility, and potential consequences of IC, including mortality [33]. Neutropenic patients, those with the most aggressive hematologic malignancies, or recipients of allogeneic stem cell transplants likely constitute a subgroup of immunocompromised patients where the challenges associated with AFT de-escalation are the most pressing for clinicians. For instance, the IDSA guidelines base the total duration of AFT in neutropenic patients not only on clinical stability, but also to the resolution of neutropenia [10]. We did not specifically record data on patients presenting with profound neutropenia at ICU admission (only those who developed profound neutropenia during ICU stay), and this specific group of patients should be the focus of future work. Finally, the limited use of formal local protocols for AFT de-escalation may have contributed to variability in prescribing practices across participating centres and may limit the generalizability of our findings to other settings.

Our study suggests that early AFT de-escalation is safe, as we observed no differences in ICU mortality, ICU length-of-stay or duration of IMV between patients with and without AFT de-escalation. These findings are consistent with previous studies conducted in non-immunocompromised cohorts [15,16,[18], [19], [20], [21]]. However, most de-escalation episodes consisted of early complete AFT discontinuation rather than spectrum reduction, and predominantly occurred in patients receiving empirical or pre-emptive treatment. Therefore, our results mainly support the feasibility of early discontinuation of AFT when IC becomes unlikely, rather than establishing the safety of early spectrum reduction in patients with proven IC. Early AFT re-initiation occurred in a minority of patients who had AFT de-escalation. Importantly, in this study AFT re-initiation was considered even in the absence of microbiologically documented IC relapse, and the reasons for re-initiation were not systematically collected. AFT re-initiation could therefore primarily reflect clinicians’ prescribing behaviour and precautionary practice in response to persistent fever, ongoing neutropenia or haemodynamic deterioration, rather than recurrence of the same documented fungal infection. This proportion of patients in whom AFT is re-started after early de-escalation can also be analysed in view of the results of the EMPIRICUS trial, which failed to demonstrate a positive impact of empirical AFT on prognostic outcomes (ICU mortality, organ failure-free days), but did show that empirical treatment was associated with a lower incidence of new proven IC [13].

As mentioned previously, the term AFT de-escalation encompasses a broad range of clinical scenarios, each associated with different perceived or demonstrated risks of IC relapse and/or complications, including mortality. A key distinction is whether AFT was initiated for proven IC (documented treatment) or probable IC (empirical or pre-emptive treatment). In cases of proven IC, the main unresolved question is whether it is safe to switch from an echinocandin to fluconazole in clinically stable patients with fluconazole-susceptible *Candida* isolates before completion of the 5 days of initial therapy recommended by the IDSA [10] and the more recent 2025 guidelines [11]. In cases of probable IC, where AFT is started empirically, the key challenge is to promptly identify the subgroup of patients who do not actually have IC (which some studies suggest may represent the majority of empirically treated patients [14]) and in whom AFT could therefore be safely discontinued altogether. While observational and interventional studies have explored the efficacy and safety of early AFT discontinuation strategies based on negative fungal biomarkers with high negative predictive value, most of these studies have excluded immunocompromised patients [15,16]. In our study, the high proportion of empirical or pre-emptive AFT, together with the relatively limited use of negative fungal cultures or biomarkers to support de-escalation, highlights an important opportunity for antifungal stewardship. This suggests that structured de-escalation protocols and more systematic use of rapid diagnostics and fungal biomarkers may help reduce unnecessary antifungal exposure in immunocompromised critically ill patients. However, decisions to discontinue empirical AFT are also likely influenced by human factors, including diagnostic uncertainty, clinicians’ perception of risk, and a potential reluctance to withdraw treatment in severely immunocompromised patients. However, our study was not designed or powered to fully explore the incidence, determinants and safety associated with early AFT de-escalation in these two clinical scenarios (spectrum reduction in proven IC vs. discontinuation in probable IC) separately, and this should be the focus of future work.

Our study has important limitations. It was a retrospective study, and further prospective multicentre studies are needed to confirm these findings. Its limited sample size precluded subgroup analyses, for instance by distinguishing between different subgroups of immunocompromised patients. Decisions to de-escalate were left at the discretion of clinicians in charge of participating patients, and adherence to local protocols or published algorithms for de-escalation [15,16] was not precisely recorded. Investigators were required to record only one primary reason for AFT de-escalation per patient, and conversely, reasons associated with AFT continuation (e.g., clinical severity, immunosuppression type, positivity of biomarkers or cultures, concerns about resistant strains, etc.) were not recorded. We therefore acknowledge that our dataset may not fully capture the complexity and multifactorial nature of clinical decision-making surrounding early AFT de-escalation. Quantitative and serial (1,3)-β-d-glucan measurements were not systematically collected, precluding assessment of biomarker kinetics or of how individual assay values influenced AFT de-escalation decisions. Data on switching to oral antifungal therapy, as recommended in some guidelines, were not systematically collected [11]. Proven and probable IC were classified according to the EORTC/MSGERC criteria predefined when the study started in 2019. The FUNDICU consensus definitions, which were published in 2024 and provide ICU-specific definitions, including more granular criteria for intra-abdominal candidiasis, could not be applied retrospectively because not all required variables had been systematically collected. Although FUNDICU was primarily developed for non-neutropenic critically ill patients without classical host factors, its application might have resulted in fewer patients being classified as having probable IC. This potential classification difference may limit the generalizability of our findings to cohorts defined using FUNDICU criteria. Importantly, given the observational design of the study, residual confounding—including confounding by indication and unmeasured factors influencing clinicians’ decisions to de-escalate treatment—cannot be excluded, and associations between AFT de-escalation and patient-centred outcomes should not be interpreted as causal. Antifungal-related toxicities, including hepatotoxicity and nephrotoxicity, were not systematically collected and could therefore not be assessed. Finally, the potential benefits of AFT de-escalation on costs and antifungal resistance were not evaluated.

## Conclusion

Early AFT de-escalation occurred in 38% of critically ill immunocompromised patients with proven or probable IC, and was not associated with adverse prognostic outcomes.

## CRediT authorship contribution statement

KJ: conceptualization, data collection, manuscript drafting, final revision of the manuscript. CB: data collection, manuscript drafting, final revision of the manuscript. LK: manuscript drafting, final revision of the manuscript. AR: manuscript drafting, final revision of the manuscript. ML: data collection, final revision of the manuscript. AS: data collection, final revision of the manuscript. JR: data collection, final revision of the manuscript. GB: data collection, final revision of the manuscript. AD: data collection, final revision of the manuscript. BS: data collection, final revision of the manuscript. JL: statistical analysis, final revision of the manuscript. SN: conceptualization, supervision, final revision of the manuscript.

## Consent for publication

Not applicable.

## Declaration of Generative AI and AI-assisted technologies in the writing process

The authors used ChatGPT (OpenAI, GPT-5) to assist with grammar and syntax during manuscript preparation. All outputs were reviewed and edited by the authors, who take full responsibility for the content of the published article.

## Funding

No specific funding was obtained for this study.

## Availability of data and materials

The datasets used and analysed during the current study are available from the corresponding author on reasonable request.

## Declaration of competing interest

No competing interests related to this manuscript.
