# Supplementary material for: Early de-escalation of antifungal treatment in critically ill immunocompromised patients with proven or probable invasive candidiasis: A retrospective multicentre study
Source: Ann Intensive Care. 2026 Aug 25;16:100139. doi: 10.1016/j.aicoj.2026.100139 (PMC13564122; doi:10.1016/j.aicoj.2026.100139)
Supplement: Supplementary file 1 [file mmc1.docx]

**Early de-escalation of antifungal treatment in critically ill immunocompromised patients with proven or probable invasive candidiasis: a retrospective multicentre study**

**Supplementary material**

**Supplementary Table 1.** Distribution of Candida species isolated from sterile and non-sterile sites

|  | **De-escalation** | |  |
| --- | --- | --- | --- |
| **Characteristics** | **No (*n*=150)** | **Yes (*n*=92)** | ***P-Va*lue** |
| **Sterile sites** |  |  | . |
| Patients with ≥1 positive sample(s) from a sterile site | 48/150 (32%) | 23/92 (25%) | 0.310 |
| *Candida albicans* | 26/48 (54.2%) | 19/23 (82.6%) | 0.028 |
| *Nakaseomyces glabratus* | 13/48 (27.1%) | 1/23 (4.3%) |  |
| *Candida parapsilosis* | 2/48 (4.2%) | 3/23 (13.0%) |  |
| *Candida tropicalis* | 2/48 (4.2%) | 0/23 (0.0%) |  |
| *Pichia kudriavzevii* | 4/48 (8.3%) | 0/23 (0.0%) |  |
| *Kluyveromyces marxianus* | 3/48 (6.3%) | 0/23 (0.0%) |  |
| *Clavispora lusitaniae* | 3/48 (6.3%) | 0/23 (0.0%) |  |
| Others | 8/48 (16.7%) | 1/23 (4.3%) |  |
| **Non-sterile sites** |  |  | . |
| Patients with ≥1 positive sample(s) from a non-sterile site | 64/150 (42.7) | 37/92 (40.2) | 0.810 |
| *Candida albicans* | 40/64 (62.5%) | 25/37 (67.6%) | 0.671 |
| *Nakaseomyces glabratus* | 13/64 (20.3%) | 7/37 (18.9%) |  |
| *Candida parapsilosis* | 4/64 (6.3%) | 5/37 (13.5%) |  |
| *Candida tropicalis* | 1/64 (1.6%) | 2/37 (5.4%) |  |
| *Pichia kudriavzevii* | 3/64 (4.7%) | 1/37 (2.7%) |  |
| *Kluyveromyces marxianus* | 4/64 (6.3%) | 1/37 (2.7%) |  |
| *Clavispora lusitaniae* | 2/64 (3.1%) | 1/37 (2.7%) |  |
| Others | 11/64 (17.2%) | 3/37 (8.1%) |  |

Values are no./total.no (%) or median (25^th^ to 75^th^ percentiles).

Sterile sites were blood cultures, cerebrospinal fluid, pleural fluid, peritoneal fluid, pericardial fluid, surgical site specimens. Non-sterile sites were skin, urine, trachea, catheter, nasal/rectal swabs.

**Supplementary Table 2.** Multivariable logistic regression analysis of predictors of AFT de-escalation

| **Predictors** | **Odds ratio**  **(95% confidence interval)** | **P-Value** |
| --- | --- | --- |
| Age (per 10-year increase) | 1.10 (0.89 to 1.35) | 0.38 |
| Male sex | 1.73 (0.97 to 3.10) | 0.063 |
| Liver cirrhosis | 0.32 (0.06 to 1.55) | 0.16 |
| Solid malignancy | 0.92 (0.43 to 1.95) | 0.82 |
| Shock | 1.67 (0.93 to 3.00) | 0.086 |
| Post-operative care | 1.76 (0.80 to 3.86) | 0.16 |

Candidate variables for the multivariable model were selected among variables associated with AFT de-escalation at a significance level of *p* < 0.20 in univariable analyses. Admission category was not considered because it conveyed overlapping information with postoperative care.

**Supplementary Table 3.** Patients’ outcomes according to AFT de-escalation type

| **28-day outcomes** | **No de-escalation**  **(*n*=150)** | **Spectrum reduction**  **(*n*=33)** | **Complete discontinuation**  **(*n*=59)** | *p*-value** |
| --- | --- | --- | --- | --- |
| **ICU mortality** |  |  |  |  |
| no./total no. (%) | 43 / 150 (28.7) | 8 / 33 (24.2) | 10 / 59 (17.0) |  |
| cHR (95% CI) | 1.00 (reference) | 0.68 (0.31 to 1.45) | 0.67 (0.33 to 1.34) | 0.37 |
| **Ventilator-free days** |  |  |  |  |
| Median (25^th^ to 75^th^ percentiles) | 16 (0 to 26) [n=149] | 9 [0 to 23] | 21 [4 to 27] |  |
| GenOR (95% CI) | 1.00 (reference) | 0.83 (0.54 to 1.27) | 1.29 (0.91 to 1.83) | 0.17 |
| **ICU length-of-stay** |  |  |  |  |
| no./total no (%)* | 82 / 150 (58.6) | 20 / 33 (60.6) | 38 / 59 (64.4) |  |
| cHR (95% CI) | 1.00 (reference) | 0.95 (0.58 to 1.56) | 1.28 (0.87 to 1.89) | 0.39 |

cHR indicates cause-specific hazard ratio, treating ICU discharge alive (for the ‘ICU mortality’ outcome) and ICU mortality (for the ‘ICU length-of-stay’ outcome) as competing risks. GenOR indicates generalized odds ratio provided for higher ventilator-free days values.

ICU: intensive care unit.

*number and percentage of ICU discharge alive.

**p-value for comparison between the three groups (calculated using Cox’s regression model or Kruskal Wallis test for ventilator-free days).

**Supplementary Figure 1.** Effect sizes of AFT-de-escalation on 28-day outcomes according to the type of invasive candidiasis (proven vs. probable IC)

Effect sizes were expressed as 1) cause-specific hazard ratio (cHR), treating ICU discharge alive (for the ‘ICU mortality’ outcome) and ICU mortality (for the ‘ICU length-of-stay’ outcome) as competing risk; 2) generalized odds ratio provided for higher ventilator-free days values. P-value for heterogeneity calculated using Z-score statistics comparing effect sizes among patients with proven versus probable IC.

ICU: intensive care unit.
